# Marine-Derived Fungal Metabolite MHO7 Promotes Breast Cancer Apoptosis as a Hippo Pathway Regulator by Modulating the YAP-TEAD Axis

**DOI:** 10.3390/md24080273

**Published:** 2026-08-06

**Authors:** Xue Ren, Linfei Wang, Yuxuan Huang, Bei Shu, Kerui Hou, Mengyao Chen, Yao Xiao, Jiahong Liang, Hao Yan, Shuaishuai Ding, Hui Qiu, Jin Lu, Kui Hong, Xin Liu

**Affiliations:** 1 Department of Radiation and Medical Oncology (Gynecological Cancers), Zhongnan Hospital of Wuhan University, School of Pharmaceutical Sciences, Wuhan University, Wuhan 430072, China; 15228477489@163.com (X.R.); 15163099203@163.com (L.W.); 2024283060057@whu.edu.cn (Y.H.); shubei2001@whu.edu.cn (B.S.); hmnb314159@163.com (K.H.); 2019303060046@whu.edu.cn (M.C.); 2025183060049@whu.edu.cn (Y.X.); 17765231847@163.com (J.L.); 2022303061012@whu.edu.cn (H.Y.); 2021203060055@whu.edu.cn (S.D.); qiuhuiznyy@whu.edu.cn (H.Q.); 15827595620@163.com (J.L.); 2 The First Affiliated Hospital and College of Clinical Medicine of Henan University of Science and Technology, Luoyang 471003, China

**Keywords:** MHO7, breast cancer, Hippo signaling pathway, TEAD, anticancer therapy

## Abstract

Breast cancer, especially triple-negative breast cancer (TNBC) and endocrine-resistant disease, remains difficult to treat because of limited effective targeted therapies. In this study, we evaluated the antitumor activity and potential mechanism of MHO7, a marine-derived ophiobolin metabolite, in ER-positive ZR-75-30 cells, tamoxifen-resistant LCC2 cells, and TNBC MDA-MB-231 models. MHO7 dose-dependently reduced cell viability, wound closure, and clonogenic growth in ZR-75-30 and LCC2 cells, with IC_50_ values of 11.53 and 10.43 μM, respectively. MHO7 also promoted apoptotic cell death, accompanied by increased reactive oxygen species accumulation and altered expression of apoptosis-related proteins, including Bcl-2 and caspase-3. N-acetyl-L-cysteine partially attenuated MHO7-induced apoptosis and YAP reduction in MDA-MB-231 cells, suggesting a contribution of oxidative stress. Molecular docking predicted that MHO7 could occupy the conserved TEAD palmitoylation pocket, and subsequent in vitro analyses showed suppression of YAP/TAZ-TEAD signaling, including reduced YAP nuclear accumulation and decreased TEAD4 expression. In an MDA-MB-231 xenograft model, MHO7 significantly inhibited tumor growth, reduced CD31-positive microvessel density, and decreased Hippo pathway-related transcriptional readouts. These findings indicate that MHO7 exerts broad antitumor activity in breast cancer models through oxidative stress-associated apoptosis and modulation of YAP/TAZ-TEAD signaling, supporting its further development as a lead compound for treatment-resistant breast cancer.

## 1. Introduction

Breast cancer remains the most commonly diagnosed cancer in women worldwide and a leading cause of cancer-related mortality [1,2,3,4]. Despite substantial advances in early detection and systemic treatment, breast cancer continues to pose a major clinical challenge because of its marked molecular heterogeneity, which affects disease progression, therapeutic response, and prognosis [5,6,7]. Clinically, breast cancers are commonly classified according to the expression of estrogen receptor (ER), progesterone receptor (PR), and human epidermal growth factor receptor 2 (HER2), broadly defining hormone receptor-positive, HER2-positive, and triple-negative disease categories [8,9,10]. Although endocrine therapy and HER2-targeted treatment have significantly improved outcomes for selected patient populations [11,12], triple-negative breast cancer (TNBC) remains a particularly aggressive subtype characterized by rapid progression, early metastasis, and limited therapeutic options [13,14,15,16,17].

Natural products have long served as an important source of anticancer agents because of their structural diversity and unique mechanisms of action [18,19]. Among these, fungal metabolites have attracted growing attention as a source of bioactive compounds with potential efficacy against refractory malignancies. MHO7, a natural product derived from the mangrove-associated fungus *Aspergillus ustus*, belongs to the ophiobolin family of fungal metabolites and has shown promising anticancer activity [20,21,22,23]. Previous studies have demonstrated that MHO7 and related compounds can affect cancer cell survival through multiple mechanisms, including estrogen receptor degradation, endoplasmic reticulum stress-associated immunogenic cell death, and mitochondria-related programmed cell death [20,21,22,23]. However, despite these encouraging findings, the molecular basis underlying the activity of MHO7 in breast cancer, particularly in treatment-resistant and aggressive subtypes, remains insufficiently defined.

Previous studies have identified the Hippo pathway as a key regulator in various cancers, including breast cancer, particularly through its downstream effector YAP/TAZ-TEAD module [24,25]. This pathway governs cell proliferation, survival, migration, and tissue homeostasis, and its dysregulation in aggressive subtypes such as triple-negative breast cancer (TNBC) contributes to uncontrolled growth and early metastasis [26,27,28,29,30]. Under normal conditions, Hippo pathway activation restrains tumorigenic signaling by promoting phosphorylation-dependent cytoplasmic sequestration of YAP and TAZ, limiting their interaction with TEAD transcription factors [30]. Aberrant activation of YAP/TAZ-driven transcription has been associated with malignant progression and poor outcome in breast cancer [31,32], with TEAD4 emerging as a critical mediator of oncogenic transcriptional programs [24,30,33]. Despite identification of multiple Hippo pathway modulators, the potential of natural products, such as MHO7, to modulate the YAP/TAZ-TEAD signaling axis in breast cancer remains largely unexplored.

Given the recognized role of YAP/TAZ-TEAD signaling in aggressive breast cancer, especially TNBC, and the continuing need for agents active in endocrine-resistant disease, we investigated the antitumor effects of MHO7 across ER-positive, tamoxifen-resistant, and triple-negative breast cancer models. We specifically asked whether the growth-inhibitory activity of MHO7 is accompanied by modulation of Hippo pathway-associated signaling in addition to apoptosis-related responses. By integrating in vitro analyses with an in vivo TNBC xenograft model, this study aimed to define the antitumor profile of MHO7 and to evaluate whether the YAP/TAZ-TEAD axis may contribute to its mechanism of action.

## 2. Results

### 2.1. MHO7 Reduces Cell Viability, Wound Closure, and Colony Formation in ZR-75-30 and LCC2 Cells

**Figure 1:**
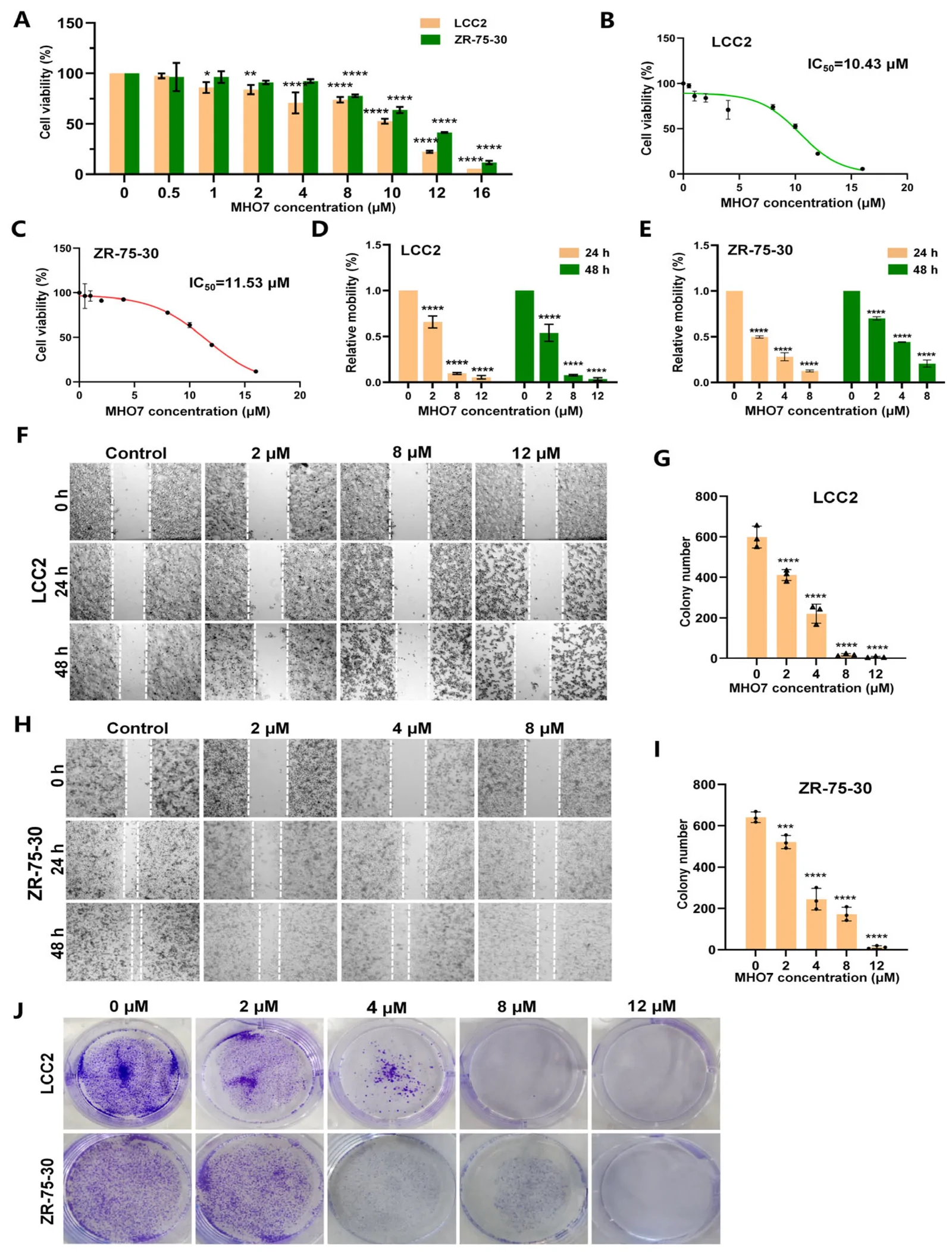
Effects of MHO7 on Viability, Wound Closure, and Proliferation Capacity of ZR-75-30 and LCC2 Cells. (**A**) Cell viability of ZR-75-30 and LCC2 cells treated with increasing concentrations of MHO7 for 48 h. (**B**,**C**) Dose–response curves showing the effects of MHO7 on the viability of LCC2 (IC_50_ = 10.43 μM) and ZR-75-30 (IC_50_ = 11.53 μM) cells. (**D**,**E**) Quantification of relative migration rate of LCC2 and ZR-75-30 cells after MHO7 treatment for 24 and 48 h. (**F**,**H**) Representative images of LCC2 cells treated with MHO7 (0, 2, 8, and 12 μM) and ZR-75-30 cells treated with MHO7 (0, 2, 4, and 8 μM) at 0, 24, and 48 h post-treatment. (**G**,**I**) Colony formation by LCC2 and ZR-75-30 cells following MHO7 treatment (0, 2, 4, 8, and 12 μM). (**J**) Representative images of colonies formed by LCC2 and ZR-75-30 cells after treatment with MHO7 (0, 2, 4, 8, and 12 μM). Data are expressed as mean ± SD (*n* = 3). Significance markers indicate comparison with the 0 μM group within the same cell line. * *p* < 0.05, ** *p* < 0.01, *** *p* < 0.001, **** *p* < 0.0001.

Treatment with MHO7 (0, 0.5, 1, 2, 4, 8, 10, 12, and 16 µM) for 48 h led to a dose-dependent decrease in cell viability in both cell lines. The half-maximal inhibitory concentration (IC_50_) values were 11.53 µM for ZR-75-30 cells and 10.43 µM for LCC2 cells, suggesting that LCC2 cells remained responsive to MHO7 despite tamoxifen resistance under the tested conditions (Figure 1A–C). Together, these results indicate that MHO7 reduces cell viability in both ER-positive and tamoxifen-resistant breast cancer cells.

Compared with the vehicle control, MHO7 reduced wound closure in both cell lines at 24 and 48 h (Figure 1D–F,H). In LCC2 cells, treatment with 2, 8, and 12 μM MHO7 progressively decreased relative wound closure, with the strongest effect observed at 12 μM after 48 h (Figure 1D,F). A similar trend was observed in ZR-75-30 cells treated with 2, 4, and 8 μM MHO7, in which residual wound gaps remained wider than those in the control group at both time points (Figure 1E,H). These data indicate that MHO7 impaired wound closure under the present experimental conditions.

Clonogenic growth was also reduced in both ZR-75-30 and LCC2 cells, as reflected by decreases in colony number and colony size relative to the control groups (Figure 1G,I). A measurable reduction in colony-forming ability was already observed at 2 µM, whereas colony formation was minimal at 12 µM in both cell lines (Figure 1J).

### 2.2. MHO7 Induces Apoptosis in ZR-75-30 and LCC2 Cells

**Figure 2:**
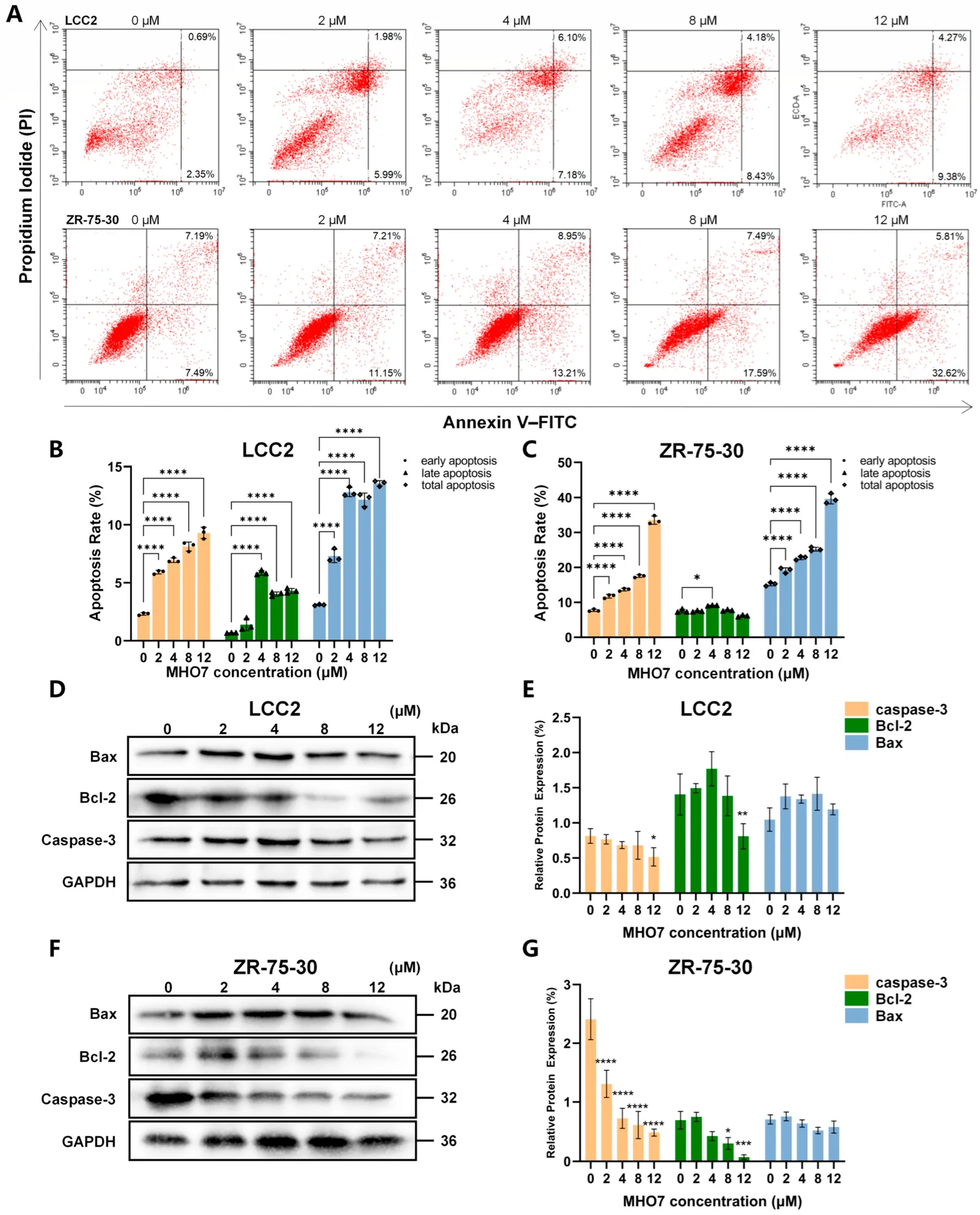
MHO7 induces apoptosis in LCC2 and ZR-75-30 cells by promoting apoptotic cell death and regulating apoptosis-related proteins. (**A**) Representative Annexin V-FITC/PI staining profiles of LCC2 and ZR-75-30 cells treated with MHO7 (0, 2, 4, 8, and 12 μM) for 48 h. The numbers in the lower right and upper right quadrants represent the percentages of early and late apoptotic cells, respectively. (**B**,**C**) Quantification of early apoptosis, late apoptosis, and total apoptosis in LCC2 and ZR-75-30 cells, respectively. (**D**,**F**) Representative protein bands of Bax, Bcl-2, caspase-3, and GAPDH in LCC2 and ZR-75-30 cells treated with MHO7 at 0, 2, 4, 8, and 12 μM for 48 h. (**E**,**G**) Quantification of Bax, Bcl-2, and caspase-3 protein expression levels in LCC2 and ZR-75-30 cells, respectively. Protein expression was normalized to GAPDH. Data are expressed as mean ± SD (*n* = 3). Significance markers indicate comparison with the 0 μM group within the same cell line. * *p* < 0.05, ** *p* < 0.01, *** *p* < 0.001, **** *p* < 0.0001.

MHO7 increased apoptotic cell populations in both ZR-75-30 and LCC2 cells, as determined by Annexin V-FITC/PI staining (Figure 2A). In LCC2 cells, both early and late apoptotic fractions increased with MHO7 treatment, leading to an overall concentration-dependent increase in total apoptosis across the tested concentration range (Figure 2B). In ZR-75-30 cells, MHO7 also increased total apoptotic cells, with the most prominent change observed in the early apoptotic population, whereas the late apoptotic fraction showed comparatively limited variation (Figure 2C). At 12 μM, the early apoptotic population reached 9.38% in LCC2 cells and 32.62% in ZR-75-30 cells, while the corresponding total apoptotic populations were 13.65% and 38.43%, respectively (Figure 2A). Together, these findings indicate that MHO7 promotes apoptotic cell death in both breast cancer cell lines, although the relative distribution of early and late apoptotic populations differed somewhat between the two models.

Changes in apoptosis-related proteins were also observed following MHO7 treatment (Figure 2D–G). In LCC2 cells, Bax levels showed relatively limited variation across the tested concentrations, whereas Bcl-2 expression decreased more evidently at higher concentrations, particularly at 12 μM. Full-length caspase-3 also showed a modest decrease after MHO7 exposure (Figure 2D,E). In ZR-75-30 cells, MHO7 treatment was accompanied by a clearer reduction in Bcl-2 and full-length caspase-3, while Bax levels remained comparatively stable overall (Figure 2F,G). Taken together, these protein changes are consistent with apoptosis-related molecular alterations following MHO7 treatment and suggest involvement of mitochondria-associated apoptotic signaling, thereby supporting the flow cytometric evidence of apoptotic cell death in both cell lines.

### 2.3. MHO7 Elevates Intracellular Reactive Oxygen Species in ZR-75-30 and LCC2 Cells

**Figure 3:**
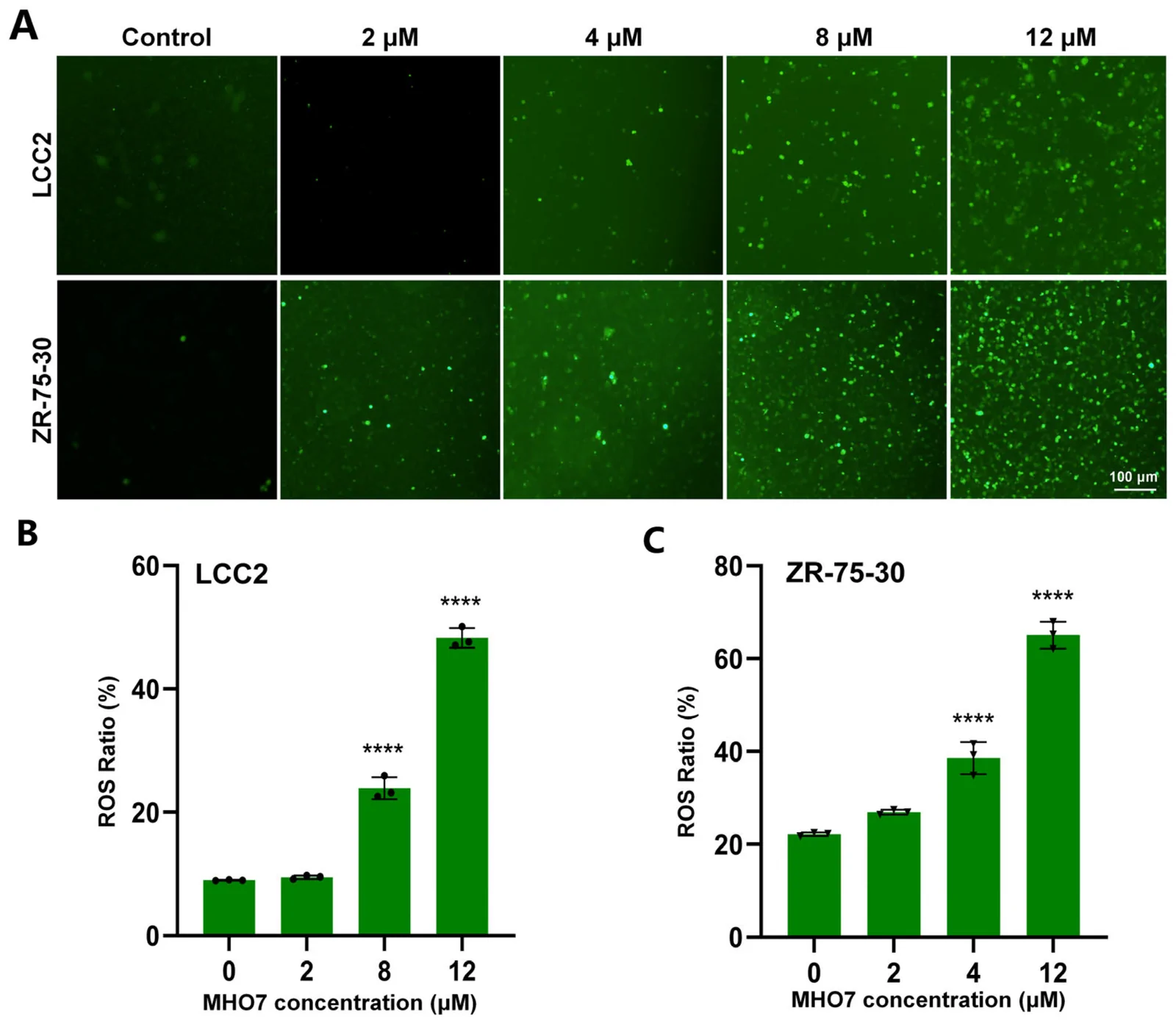
MHO7 promotes reactive oxygen species (ROS) accumulation in LCC2 and ZR-75-30 cells. (**A**) Representative fluorescence microscopy images showing ROS production (indicated by enhanced green DCF fluorescence) in LCC2 and ZR-75-30 cells treated with the indicated concentrations of MHO7 (0, 2, 4, 8, and 12 μM) for 48 h. Scale bar: 100 μm. (**B**,**C**) Quantification of intracellular ROS levels in LCC2 and ZR-75-30 cells, respectively. The corresponding fluorescence intensity distribution profiles are shown in Figure S1 and Figure S2, respectively. Data are expressed as mean ± SD (*n* = 3). Significance markers indicate comparison with the 0 μM group within the same cell line. **** *p* < 0.0001.

Intracellular reactive oxygen species (ROS) levels were measured in LCC2 and ZR-75-30 cells after MHO7 treatment. Fluorescence microscopy showed that MHO7 treatment increased DCF fluorescence intensity in both LCC2 and ZR-75-30 cells compared with the corresponding control groups (Figure 3A). In LCC2 cells, stronger fluorescence signals were observed at 8 and 12 µM, whereas in ZR-75-30 cells the increase became more evident at 4 and 12 µM, with the strongest signal detected at 12 µM.

In LCC2 cells, the proportion of DCF-positive cells remained close to baseline at 2 µM but increased markedly at 8 and 12 µM (Figure 3B and Figure S1). In ZR-75-30 cells, ROS levels also increased following MHO7 treatment, with more evident elevation at 4 and 12 µM than at 2 µM (Figure 3C and Figure S2). The corresponding flow cytometry histograms showed a rightward shift in DCF fluorescence intensity together with an expansion of the DCF-positive population, particularly at higher concentrations (Figures S1 and S2). Together, these findings indicate that MHO7 promotes intracellular ROS accumulation in both ER-positive and tamoxifen-resistant breast cancer cells, particularly at higher concentrations.

### 2.4. N-Acetyl-L-Cysteine Attenuates MHO7-Induced Apoptosis in MDA-MB-231 Cells

**Figure 4:**
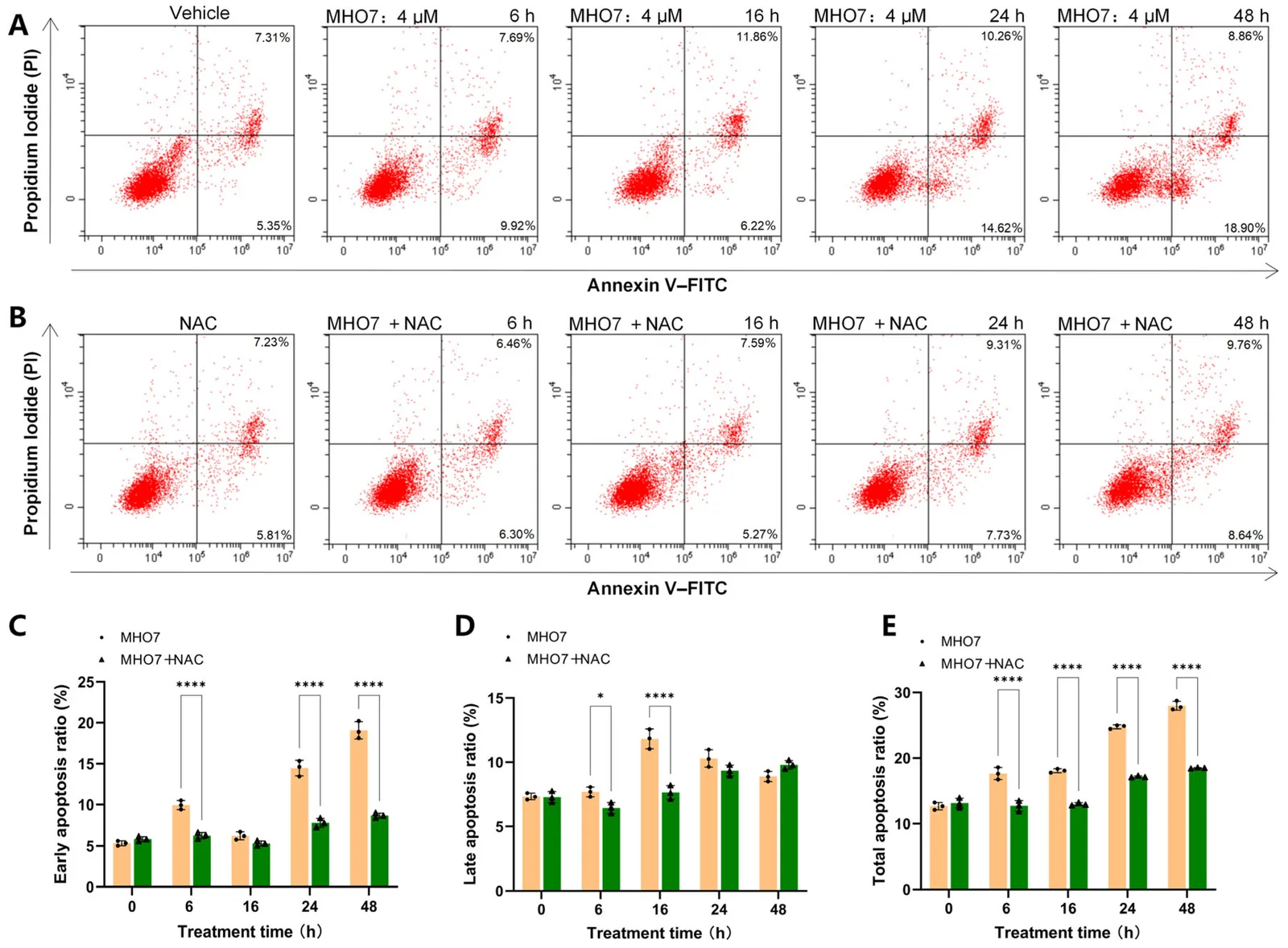
N-Acetyl-L-cysteine attenuates MHO7-induced apoptosis in MDA-MB-231 cells. (**A**) Representative Annexin V-FITC/PI staining profiles of MDA-MB-231 cells treated with vehicle control (DMSO) or 4 μM MHO7 for 6, 16, 24, and 48 h. (**B**) Representative Annexin V-FITC/PI staining profiles of MDA-MB-231 cells treated with 4 mM N-acetyl-L-cysteine (NAC) alone or co-treated with 4 μM MHO7 and 4 mM NAC for 6, 16, 24, and 48 h. The numbers in the lower right and upper right quadrants indicate the percentages of early apoptotic and late apoptotic cells, respectively. (**C**–**E**) Quantification of early apoptotic, late apoptotic, and total apoptotic populations, respectively. Total apoptosis was calculated as the sum of early and late apoptosis. Data are expressed as mean ± SD (*n* = 3). Significance markers indicate pairwise comparisons between the corresponding conditions in (**A**,**B**): vehicle versus NAC alone at 0 h, and MHO7 versus MHO7 plus NAC at each matched treatment time. * *p* < 0.05, **** *p* < 0.0001.

NAC co-treatment attenuated MHO7-induced apoptotic cell death in MDA-MB-231 cells (Figure 4A,B). MHO7 increased total apoptotic cell populations over time, whereas the corresponding populations were reduced in the presence of NAC. Quantitative analysis showed that NAC significantly decreased the early apoptotic population at 6, 24, and 48 h, while the difference at 16 h was comparatively limited (Figure 4C). Late apoptosis was significantly reduced by NAC co-treatment at both 6 and 16 h (Figure 4D). Total apoptosis was also significantly lower in the presence of NAC at 6, 16, 24, and 48 h (Figure 4E). NAC alone did not significantly alter the basal apoptotic level compared with the vehicle control. Together, these findings indicate that NAC attenuates MHO7-induced apoptosis in MDA-MB-231 cells, suggesting that oxidative stress may contribute to the apoptotic response to MHO7.

### 2.5. MHO7 Modulates the Hippo Signaling Pathway In Vitro

**Figure 5:**
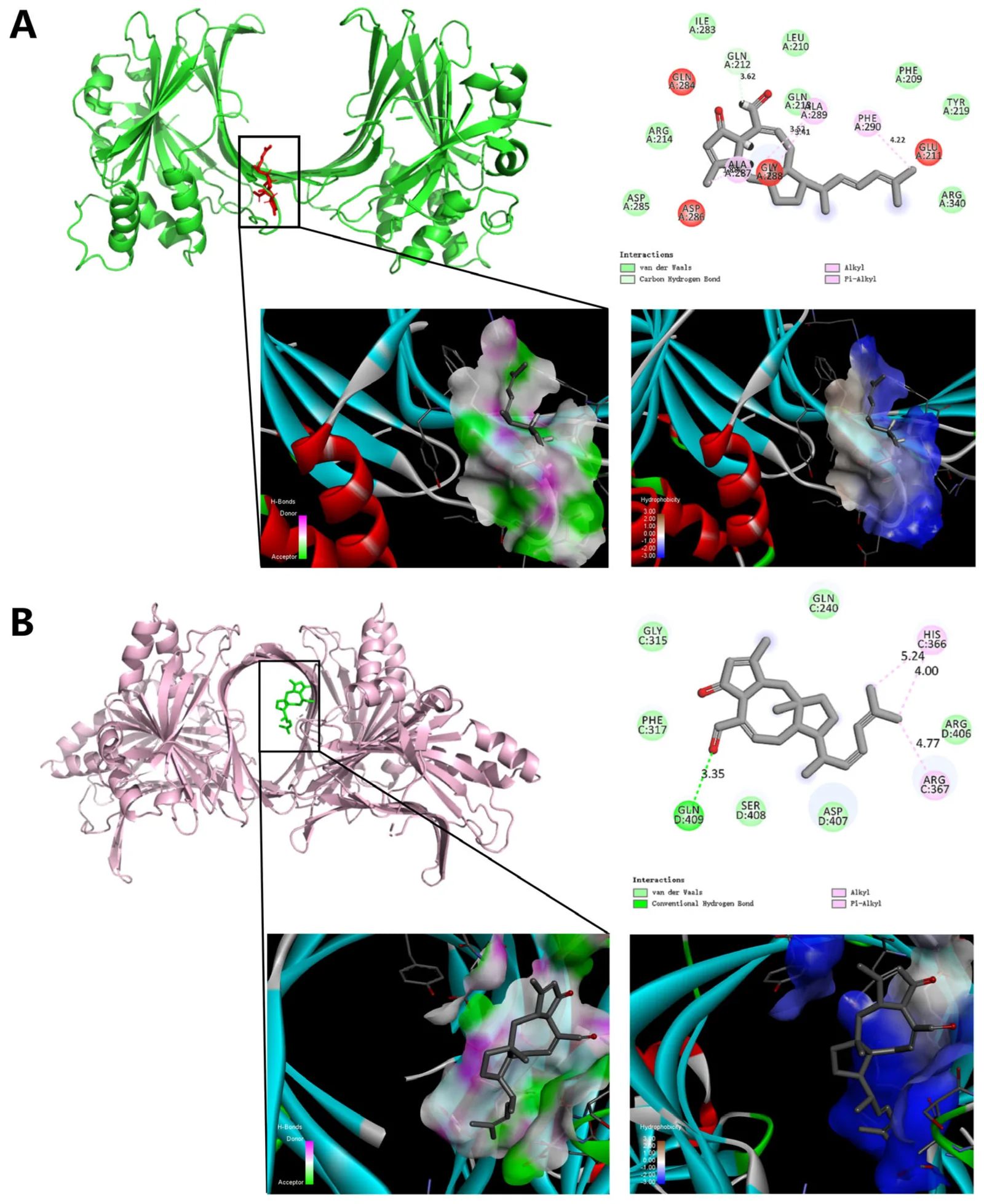
Docking Analysis of MHO7 Binding to YAP-TEAD and TEAD3. (**A**) Molecular docking simulation of MHO7 binding to the hYAP-hTEAD complex (PDB: 3KYS). The panel illustrates the overall 3D structure with a zoomed-in surface view of the binding pocket (**left**), and a 2D diagram of intermolecular interactions (**right**). Residues highlighted in red represent potential unfavorable contacts with the ligand. (**B**) Molecular docking simulation of MHO7 binding to the palmitoylated TEAD3 pocket (PDB: 5EMW). Similar to (**A**), the 3D binding mode with a zoomed-in surface view (**left**) and the 2D interaction map (**right**) are displayed.

MHO7 was predicted to interact with the hYAP-hTEAD (PDB: 3KYS) complex as well as the palmitoylated TEAD3 pocket (PDB: 5EMW), with the best docking affinities of −4.7 kcal/mol and −5.7 kcal/mol, respectively (Tables S3 and S4). In the hYAP-hTEAD model, the predicted interactions were mainly mediated by van der Waals contacts together with carbon hydrogen bond and alkyl/π-alkyl interactions (Figure 5A). By comparison, docking to the palmitoylated TEAD3 structure yielded a lower predicted binding energy and suggested that MHO7 could be accommodated within the conserved hydrophobic pocket, with one conventional hydrogen bond and multiple hydrophobic interactions (Figure 5B). These in silico results suggest that the TEAD palmitoylation pocket may represent a potentially relevant site for further investigation and provided a rationale for examining Hippo pathway-related proteins experimentally.

**Figure 6:**
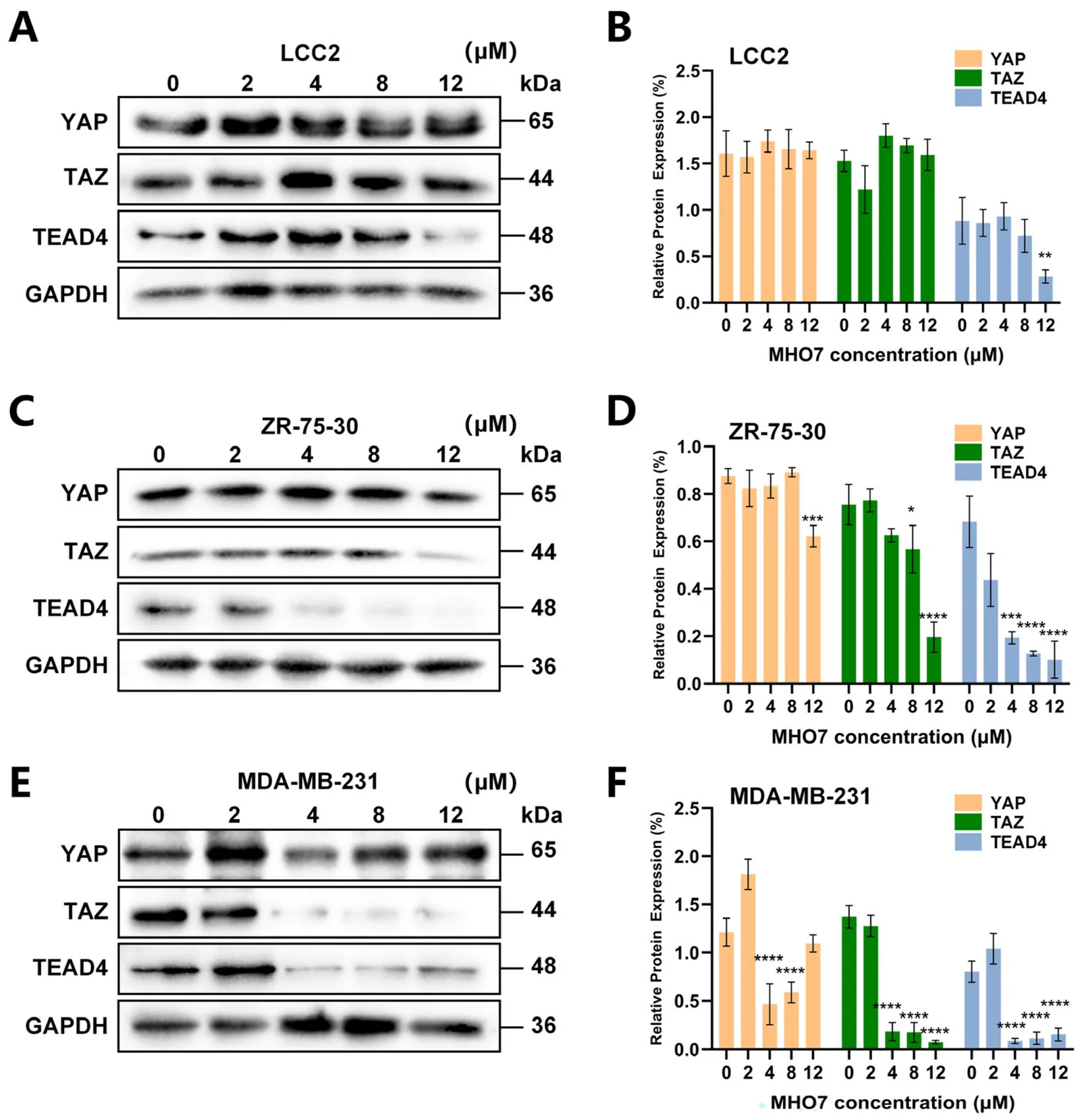
Effects of MHO7 on Hippo Pathway-Related Proteins in LCC2, ZR-75-30, and MDA-MB-231 Cells. (**A**,**C**,**E**) Representative protein bands of YAP, TAZ, TEAD4, and GAPDH in LCC2, ZR-75-30, and MDA-MB-231 cells treated with MHO7 at 0, 2, 4, 8, and 12 μM for 48 h. (**B**,**D**,**F**) Quantification of YAP, TAZ, and TEAD4 protein expression levels in LCC2, ZR-75-30, and MDA-MB-231 cells, respectively. Protein expression was normalized to GAPDH. Data are expressed as mean ± SD (*n* = 3). Significance markers indicate comparison with the 0 μM group within the same cell line. * *p* < 0.05, ** *p* < 0.01, *** *p* < 0.001, **** *p* < 0.0001.

Hippo pathway-related protein expression was altered by MHO7 in a cell type-dependent manner (Figure 6A–F). In LCC2 cells, YAP and TAZ levels showed relatively limited overall reduction across the tested concentrations, whereas TEAD4 expression decreased more clearly at 12 μM (Figure 6A,B). In ZR-75-30 cells, YAP was reduced at higher concentrations, while TEAD4 showed a marked decrease beginning at 4 μM and remained low at 8–12 μM; TAZ displayed a more moderate downward trend (Figure 6C,D). In MDA-MB-231 cells, MHO7 induced the broadest protein changes, with TAZ and TEAD4 markedly reduced from 4 μM onward, whereas YAP showed an early increase at 2 μM followed by lower levels at 4 and 8 μM relative to baseline (Figure 6E,F). Overall, these findings suggest that MHO7 is associated with suppression of Hippo pathway-related signaling at the protein level, with a more pronounced effect observed in ZR-75-30 and MDA-MB-231 cells, particularly in the TNBC model.

**Figure 7:**
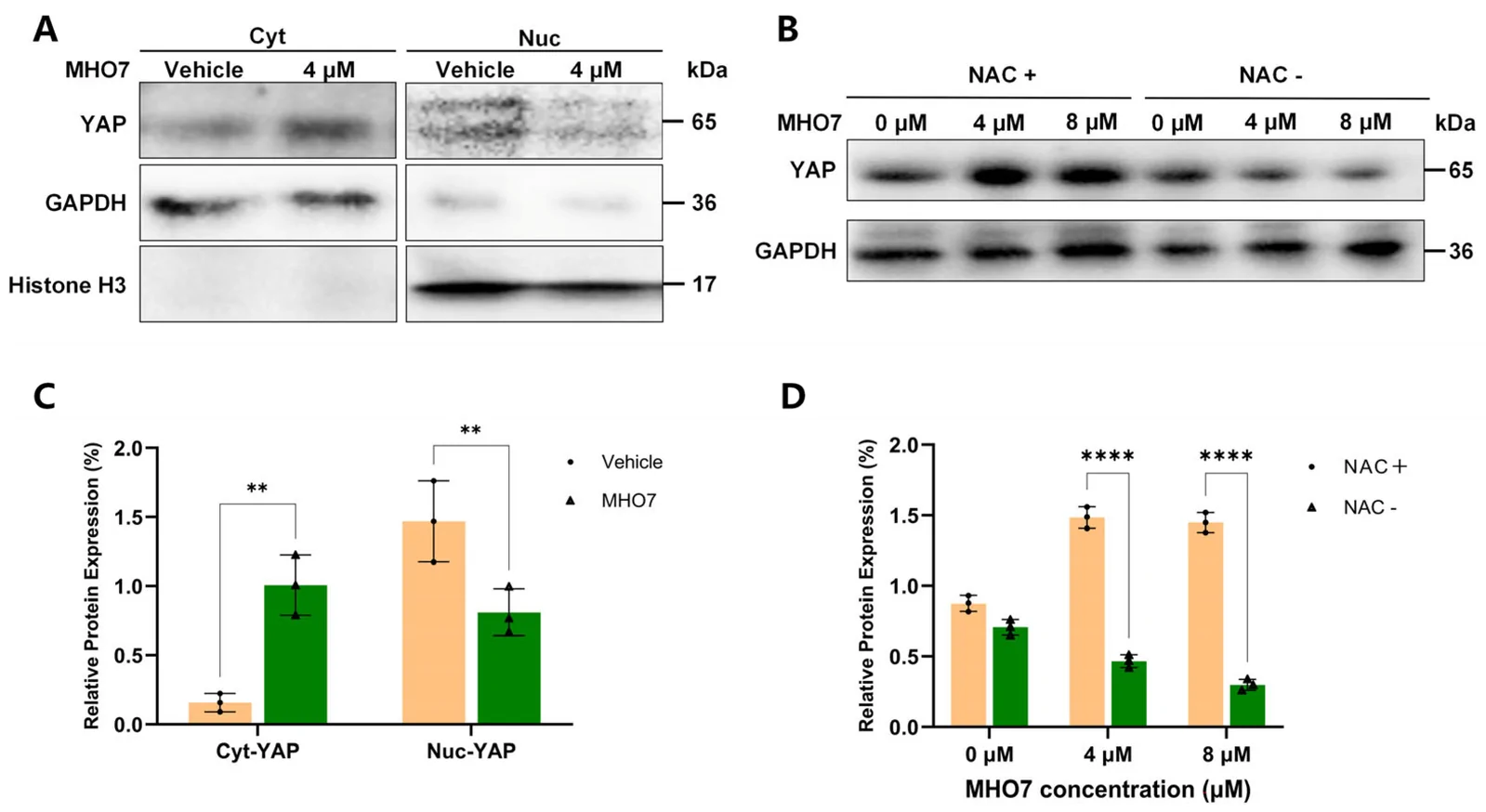
MHO7 alters YAP subcellular distribution and NAC counteracts MHO7-associated YAP reduction in MDA-MB-231 cells. (**A**) Representative protein bands of YAP in cytoplasmic (Cyt) and nuclear (Nuc) fractions from MDA-MB-231 cells treated with vehicle or 4 μM MHO7. GAPDH and Histone H3 were used as cytoplasmic and nuclear fraction markers, respectively. (**B**) Representative protein bands of YAP and GAPDH in MDA-MB-231 cells exposed to the indicated concentrations of MHO7 (0, 4, and 8 μM) in the presence or absence of 4 mM N-acetyl-L-cysteine (NAC). (**C**) Quantification of cytoplasmic and nuclear YAP protein levels corresponding to (**A**). Cytoplasmic YAP was normalized to GAPDH, whereas nuclear YAP was normalized to Histone H3. (**D**) Quantification of YAP protein expression levels corresponding to (**B**), normalized to GAPDH. Data are expressed as mean ± SD (*n* = 3). Significance markers in (**C**) indicate comparisons between vehicle- and MHO7-treated groups within the corresponding subcellular fraction. Significance markers in (**D**) indicate pairwise comparisons between NAC-positive and NAC-negative conditions at the same MHO7 concentration. ** *p* < 0.01, **** *p* < 0.0001.

Subcellular fractionation analysis further showed that MHO7 altered the intracellular distribution of YAP in MDA-MB-231 cells (Figure 7A,C). Cytoplasmic YAP levels were significantly increased following MHO7 treatment, whereas nuclear YAP levels were significantly reduced, consistent with decreased nuclear accumulation and increased cytoplasmic retention of YAP. In parallel, NAC co-treatment partially preserved YAP protein abundance in MHO7-treated cells (Figure 7B,D). In the absence of NAC, YAP levels decreased with increasing MHO7 concentrations, whereas substantially higher YAP levels were maintained in the presence of NAC at 4 and 8 μM MHO7. Together, these findings indicate that MHO7 alters the subcellular distribution of YAP and reduces its protein abundance, while NAC partially counteracts the MHO7-associated loss of YAP, suggesting that oxidative stress may contribute to YAP regulation in response to MHO7.

### 2.6. MHO7 Suppresses TNBC Xenograft Growth and Alters Hippo-Related Markers In Vivo

**Figure 8:**
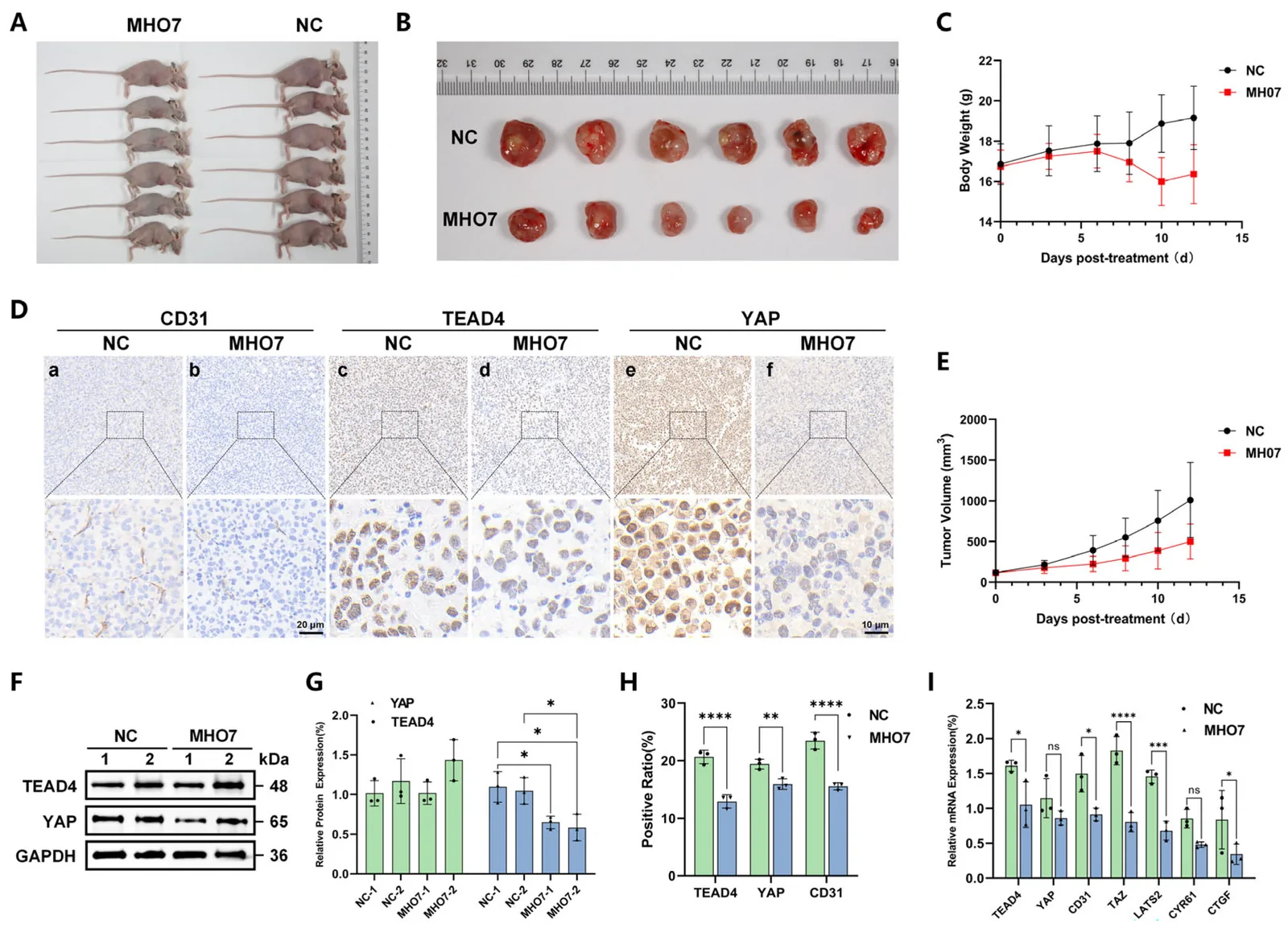
Effects of MHO7 on tumor growth and Hippo pathway-related proteins in vivo. (**A**) Photographs of mice at the end of administration. (**B**) Pictures of mouse tumors at the end of drug administration. (**C**) Body weight changes during treatment (*n* = 6 mice per group). (**D**) IHC staining of CD31, TEAD4, and YAP in tumor sections, showing reduced TEAD4/YAP staining and decreased microvessel density after MHO7 treatment. Staining details: Blue, nuclei; Brown, target proteins. Magnification: CD31, 60×; TEAD4 and YAP, 150×. Scale bars: 20 μm (CD31) and 10 μm (TEAD4/YAP). (**E**) Tumor volume changes during treatment (*n* = 6 mice per group). (**F**) Representative protein bands of TEAD4, YAP, and GAPDH in tumor tissues from the NC and MHO7 groups. (**G**) Quantification of TEAD4 and YAP protein expression levels normalized to GAPDH. (**H**) Semi-quantification of IHC analysis presented as the percentage of TEAD4-, YAP-, or CD31-positive cells (DAB-positive) in tumor sections, supporting reduced TEAD4/YAP activation (less nuclear staining) and decreased angiogenesis after MHO7 treatment. Protein expression levels were assessed using the average optical density (AOD). (**I**) RT-qPCR analysis of mRNA expression levels for Hippo pathway components (TEAD4, YAP, TAZ, LATS2), downstream targets (CYR61, CTGF), and the angiogenesis marker CD31. Data are expressed as mean ± SD (*n* = 3). Significance markers indicate comparison with the NC group. * *p* < 0.05, ** *p* < 0.01, *** *p* < 0.001, **** *p* < 0.0001. ns: no significance.

In the MDA-MB-231 xenograft model, MHO7 treatment suppressed tumor growth in vivo, as reflected by smaller excised tumors and reduced tumor growth curves compared with the control group (Figure 8A,B). By the end of the treatment period, tumor volume and tumor weight were reduced by 44.68% and 58.31%, respectively, in the MHO7 group relative to the NC group (Figure 8C,E). Together, these findings support the in vivo antitumor activity of MHO7 in this TNBC model.

In control tumors, TEAD4 immunoreactivity was observed predominantly in the nucleus, with strong brown DAB staining indicating prominent nuclear localization (Figure 8D). Following MHO7 treatment, nuclear TEAD4 staining was clearly reduced, whereas cytoplasmic staining became more apparent, suggesting an altered subcellular distribution of TEAD4 in vivo. A similar pattern was observed for YAP. In control tumors, YAP staining showed substantial overlap with the nuclear compartment, while in MHO7-treated tumors, nuclear YAP staining was weaker and a larger proportion of the signal appeared to be retained in the cytoplasm. Semi-quantitative analysis of IHC staining further supported these changes, showing reduced TEAD4- and YAP-positive signals in the MHO7 group compared with the NC group (Figure 8H). In addition, CD31 staining was also reduced in tumor tissues from the MHO7-treated group, suggesting decreased tumor-associated vascularization following treatment. In parallel, RT-qPCR analysis showed reduced mRNA expression of several Hippo pathway-related genes and downstream targets in tumor tissues from the MHO7-treated group (Figure 8I). Together, these observations are consistent with altered TEAD4 and YAP localization, along with decreased tumor-associated vascularization, following MHO7 treatment in vivo.

In agreement with the immunohistochemical findings, YAP protein abundance was reduced in tumor tissues from the MHO7 group, whereas total TEAD4 protein levels showed only limited overall change by Western blotting (Figure 8F,G). At the transcript level, RT-qPCR analysis showed decreased mRNA expression of TEAD4, CD31, TAZ, LATS2, and CTGF in the MHO7-treated group, while YAP and CYR61 did not differ significantly between groups (Figure 8I). Taken together, these data indicate that MHO7 alters Hippo pathway-related molecular readouts in vivo. Although total TEAD4 protein abundance was not markedly reduced, the decrease in nuclear TEAD4 staining together with the downregulation of several pathway-related transcripts is consistent with reduced TEAD4/YAP-associated signaling activity in tumor tissues.

## 3. Discussion

Marine-derived natural products remain an important source of structurally diverse compounds with potential utility in cancer therapy [18,19]. MHO7, an ophiobolin-related metabolite derived from a marine fungus, has previously been reported to exhibit antitumor activity in breast cancer models through mechanisms including estrogen receptor degradation and immunogenic cell death [20,34,35]. In addition, prior pharmacokinetic and toxicity studies support the feasibility of further developing MHO7 as a bioactive lead compound [36]. In the present study, we further extend the antitumor profile of MHO7 by showing that it reduces cell viability, delays wound closure, suppresses clonogenic growth, and promotes apoptotic cell death in ER-positive and tamoxifen-resistant breast cancer cells, while also inhibiting tumor growth in a TNBC xenograft model. These antitumor effects are associated with modulation of Hippo pathway-related signaling, with the most evident pathway-associated changes observed in the TNBC setting, further suggesting that YAP/TAZ-TEAD signaling may contribute to the observed cellular responses.

A major finding of this study is that MHO7 was associated with attenuation of YAP/TAZ-TEAD-related signaling in breast cancer models. In our in vitro experiments, MHO7 altered YAP, TAZ, and TEAD4 protein expression in a cell type-dependent manner, with broader effects observed in MDA-MB-231 cells than in ZR-75-30 or LCC2 cells. In vivo, MHO7 treatment reduced nuclear TEAD4 and YAP staining, decreased YAP protein abundance, and downregulated several Hippo pathway-related transcripts in tumor tissues, consistent with reduced YAP/TAZ-TEAD-associated signaling after MHO7 treatment [37,38]. In addition, molecular docking suggested that the TEAD palmitoylation pocket may represent a potentially relevant site for further investigation, although these in silico observations should be interpreted as hypothesis-generating rather than definitive evidence of direct target engagement [39,40,41,42,43].

In parallel with these pathway-related changes, MHO7 promoted apoptotic cell death and intracellular ROS accumulation in ER-positive and tamoxifen-resistant breast cancer cells. Annexin V/PI staining showed increased apoptotic populations in both models, while immunoblotting demonstrated reduced Bcl-2 and full-length caspase-3, together with relatively limited changes in Bax. These data support apoptosis-associated molecular remodeling and suggest involvement of mitochondria-associated apoptotic signaling [44,45]. The increase in intracellular ROS further indicates that oxidative stress may contribute to the cellular response to MHO7 [46,47,48]. Notably, NAC intervention attenuated MHO7-induced apoptosis in MDA-MB-231 cells and partially preserved YAP protein abundance, providing additional support for a contribution of oxidative stress to these responses. This interpretation is broadly consistent with previous studies showing that ophiobolin-related compounds can preferentially affect cancer cell survival under conditions of altered redox homeostasis and mesenchymal-like stress vulnerability [21,22,46,47,49]. Nevertheless, the present data do not fully establish the causal hierarchy among ROS accumulation, apoptosis, and Hippo pathway modulation, and further mechanistic studies are required to define their interrelationships.

The in vivo findings further strengthen the biological relevance of MHO7 in TNBC. In the MDA-MB-231 xenograft model, MHO7 suppressed tumor growth, reduced CD31 staining, and altered TEAD4/YAP localization in tumor tissues. Because YAP/TAZ-TEAD signaling is known to support tumor growth, invasion, and microenvironmental adaptation in aggressive breast cancer [30,31,32], the combined IHC, Western blot, and qPCR findings suggest that pathway suppression may contribute to the antitumor activity observed in vivo. However, the molecular data also indicated that the response of individual pathway components is not entirely uniform. For example, total TEAD4 protein abundance in tumor tissues was not markedly reduced despite reduced nuclear staining and lower TEAD4 mRNA levels. This pattern suggests that MHO7 may influence TEAD4-associated signaling not only at the level of expression, but also through altered subcellular distribution and downstream transcriptional output. Such complexity is not inconsistent with prior reports that TEAD4 biology may be influenced by isoform usage, context-dependent regulation, and subcellular localization [24,33].

Several limitations should be acknowledged. The docking results provide only predictive support and do not establish direct target engagement of MHO7 with TEAD proteins. Therefore, biochemical or functional validation, such as CETSA, SPR, or YAP/TEAD reporter assays, will be required in future studies. Second, although NAC intervention provided functional evidence linking oxidative stress to MHO7-induced apoptosis and YAP regulation, the causal hierarchy among ROS accumulation, apoptosis, and Hippo pathway modulation remains to be fully established. Finally, because the in vivo findings were obtained in a single TNBC xenograft model, additional studies will be needed to determine the generalizability of these effects across other breast cancer contexts. Despite these limitations, the combined phenotypic, molecular, and in vivo findings support MHO7 as a marine-derived lead compound for further investigation in difficult-to-treat breast cancer.

**Figure 9:**
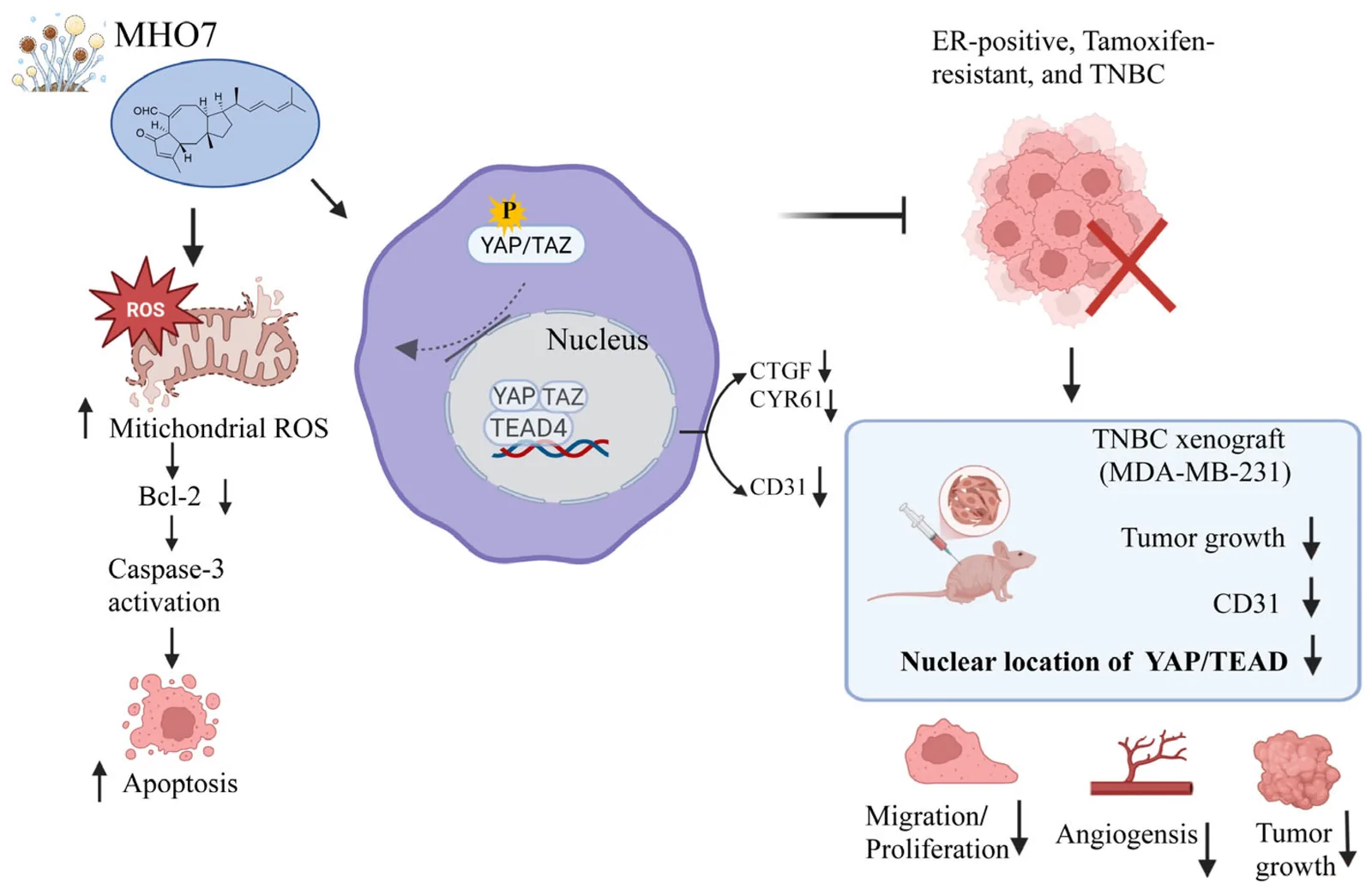
Proposed working model of the antitumor effects of MHO7 in breast cancer. MHO7 promotes intracellular ROS accumulation and apoptosis-related changes, while attenuating YAP/TAZ-TEAD signaling in breast cancer models. These effects are associated with reduced proliferation, migration, angiogenesis, and tumor growth, including suppression of TNBC xenograft growth accompanied by decreased CD31 expression and reduced nuclear YAP/TEAD4 localization. This schematic summarizes the potential actions of MHO7 based on the present findings. Arrows indicate activation or the direction of an effect, blunt-ended lines indicate inhibition, and upward or downward arrows indicate increased or decreased expression or activity, respectively. Curved arrows indicate that MHO7 inhibits the nuclear translocation of YAP/TAZ, and “P” denotes phosphorylation.

To integrate the findings from our in vitro and in vivo experiments, we propose a working model summarizing the potential actions of MHO7 in breast cancer (Figure 9). Based on the present data, MHO7 appears to exert antitumor effects through two associated processes. First, MHO7 promotes intracellular ROS accumulation and apoptotic cell death, supporting involvement of oxidative stress-associated, mitochondria-related apoptotic signaling [44,45,46,47,49]. Second, MHO7 is associated with modulation of Hippo pathway-related signaling, as reflected by altered YAP/TAZ/TEAD4 expression in vitro, reduced nuclear TEAD4 and YAP staining in vivo, and decreased expression of several pathway-related transcripts. These changes are consistent with reduced YAP/TAZ-TEAD-associated transcriptional output and may contribute to the observed decreases in tumor growth, wound closure, clonogenic outgrowth, and tumor-associated vascularization. Thus, Figure 9 should be viewed as a framework for interpretation rather than a definitive mechanistic scheme, and future studies will be needed to define the direct molecular target(s) of MHO7 and the relationship between ROS accumulation and Hippo pathway modulation.

## 4. Materials and Methods

### 4.1. Cell Lines and Reagents

The human breast cancer cell lines ZR-75-30 (ER+), LCC2 (tamoxifen-resistant), and MDA-MB-231 (TNBC) were obtained from the Life Sciences Institute, Wuhan University. MHO7 was synthesized in-house and confirmed to have >95% purity by high-performance liquid chromatography (HPLC). ZR-75-30 and LCC2 cells were cultured in RPMI-1640 medium (MA0548, MeilunBio, Dalian, China), while MDA-MB-231 cells were cultured in Leibovitz’s L-15 medium (MA0547, MeilunBio, Dalian, China). All media were supplemented with 10% fetal bovine serum (PWL217–4, MeilunBio, Dalian, China) and 1% penicillin-streptomycin (C0222, Beyotime Biotechnology, Shanghai, China). ZR-75-30 and LCC2 cells were maintained at 37 °C in a humidified incubator with 5% CO_2_, whereas MDA-MB-231 cells were maintained at 37 °C without CO_2_ supplementation.

### 4.2. Cell Viability Assay (CCK-8)

Cell viability was assessed using a CCK-8 assay. Cells were seeded in 96-well plates at 8 × 10^3^ cells/well and allowed to adhere overnight. Cells were treated with MHO7 (0, 0.5, 1, 2, 4, 8, 10, 12, and 16 μM) for 48 h (100 μL/well), followed by the addition of CCK-8 reagent (10 μL/well) (C0038, Beyotime Biotechnology, Shanghai, China) for 1 h. Absorbance was measured at 450 nm using a microplate reader (Spark 10M, Tecan, Männedorf, Switzerland). The half-maximal inhibitory concentration (IC_50_) values were calculated via non-linear regression analysis of the dose–response curves using GraphPad Prism software (v9.5.0, San Diego, CA, USA).

### 4.3. Wound Healing Assay

Cells were seeded in 6-well plates at 3–5 × 10^5^ cells/well and cultured until reaching approximately 90% confluence. An artificial wound was created by generating a linear scratch with a sterile pipette tip. Subsequently, cells were treated with MHO7 at specified concentrations (LCC2: 0, 2, 8, and 12 μM; ZR-75-30: 0, 2, 4, and 8 μM) for 48 h. Images were captured at 0, 24, and 48 h from the same fields. Wound width was quantified in ImageJ (v1.54f; National Institutes of Health, Bethesda, MD, USA), and the percentage of wound closure was calculated as (W_0_ − W_t_)/W_0_ × 100%, where W_0_ and W_t_ represent wound width at 0 h and at 24/48 h, respectively. The measurements were averaged from at least three different fields for each condition to ensure accuracy.

### 4.4. Colony Formation Assay

To evaluate clonogenic survival, cells were seeded in 6-well plates at a density of 1 × 10^3^ cells/well and allowed to adhere overnight. The cells were treated with MHO7 (0, 2, 4, 8, and 12 μM) for 72 h, after which the drug-containing medium was replaced with drug-free complete medium (2 mL/well). Cells were then cultured for an additional 10 days. Colonies were washed with phosphate-buffered saline (PBS) (BL302A, Biosharp, Beijing, China), fixed with 4% paraformaldehyde, stained with 0.1% crystal violet for 15–20 min, rinsed, air-dried, photographed, and counted. Colony counts were obtained from at least three independent experiments, and colony size was also quantified.

### 4.5. Flow Cytometry for Apoptosis

Apoptosis was quantified by flow cytometry using an Annexin V-FITC (C1062S-1, Beyotime, Shanghai, China)/propidium iodide (PI) staining assay (C1062S-3, Beyotime, Shanghai, China). For concentration-dependent apoptosis analysis, LCC2 and ZR-75-30 cells were treated with the indicated concentrations of MHO7 for 48 h. For antioxidant intervention analysis, MDA-MB-231 cells were pretreated with 4 mM N-acetyl-L-cysteine (NAC) (S0077, Beyotime, Shanghai, China) for 1 h, after which the medium was replaced with fresh medium containing 4 mM NAC and 4 μM MHO7, and the cells were further incubated for 6, 16, 24, or 48 h. Corresponding MHO7-treated groups received 4 μM MHO7 without NAC for the same durations, and vehicle- and NAC-treated cells were included as controls. Following treatment, cells were harvested, washed with PBS, and resuspended in binding buffer (C1062S-2, Beyotime, Shanghai, China) for Annexin V-FITC/PI staining. Samples were analyzed on a flow cytometer with at least 10,000 events acquired per sample, and data were processed using CytExpert (v2.4.0.28, Beckman Coulter, Brea, CA, USA). Early apoptosis was defined as Annexin V-positive/PI-negative, and late apoptosis as Annexin V-positive/PI-positive; total apoptosis was calculated as the sum of early and late apoptotic populations. Each experiment was performed in triplicate.

### 4.6. Western Blot Analysis

Total protein was extracted using lysis buffer supplemented with Phenylmethylsulfonyl fluoride (PMSF) (ST507-10mL, Beyotime Biotechnology, Haimen, China) on ice for 30 min. Protein concentrations were measured by BCA assay. Equal amounts of protein were denatured at 95 °C for 10 min, separated by SDS-PAGE, and transferred to polyvinylidene fluoride membranes (PVDF). Membranes were blocked with 5% skim milk for 1 h and incubated with primary antibodies overnight at 4 °C. The primary antibodies used in this study are detailed in Table S1. Membranes were then incubated with HRP-conjugated secondary antibodies (1:10,000, RGAR001, Proteintech, Wuhan, China) for 1 h at room temperature. Signals were detected by enhanced chemiluminescence (ECL) and imaged using a chemiluminescence imager.

### 4.7. Detection of Reactive Oxygen Species by Fluorescence Microscopy

Cells were incubated with a 10 μM 2′,7′-Dichlorodihydrofluorescein diacetate (DCFH-DA) working solution (diluted 1:1000 in serum-free medium; 2 mL per well in 6-well plates; 040-100T, Beyotime Biotechnology, Shanghai, China) at 37 °C for 20–40 min. Cells were then washed several times with serum-free medium to minimize nonspecific fluorescence and immediately imaged using a fluorescence microscope (Olympus IX73DP80, Olympus Corporation, Tokyo, Japan) with a green fluorescence emission filter. All images were acquired using identical exposure settings across groups.

### 4.8. Detection of Reactive Oxygen Species by Flow Cytometry

Cells were harvested and resuspended in PBS at a density of 1–5 × 10^5^ cells/mL, followed by incubation with DCFH-DA (1:1000) for 40 min at 37 °C in the dark. After being washed three times with PBS, cells were resuspended in 500 μL of PBS and analyzed on a CytoFLEX S flow cytometer (Beckman Coulter) using the FITC channel (excitation/emission: 510/527 nm). Cells treated with Rosup (040-100T, Beyotime Biotechnology, Shanghai, China) were utilized as a positive control. Data were analyzed using CytExpert software (v2.4.0.28).

### 4.9. Molecular Docking Study

Molecular docking was performed using AutoDock Vina (v1.1.2) to predict the binding modes of MHO7. The crystal structures of the human YAP-TEAD complex (PDB: 3KYS) and palmitoylated TEAD3 YAP-binding domain (PDB: 5EMW) were obtained from the Protein Data Bank (PDB). Protein structures were prepared in AutoDockTools (v1.5.7), involving the removal of water molecules and heteroatoms, the addition of polar hydrogens, and assigning Gasteiger charges. The 2D structure of MHO7 was sketched in ChemDraw (version 25.0; Revvity Signals Software, Waltham, MA, USA), converted to a minimized 3D structure in Chem3D, and exported in PDBQT format. Docking results, including binding affinities and intermolecular interactions, were visualized and analyzed using PyMOL (v3.0.3) and Discovery Studio Visualizer (v21.1.0.20298).

### 4.10. Cytoplasmic and Nuclear Protein Fractionation

Cytoplasmic and nuclear protein fractions were prepared from MDA-MB-231 cells treated with vehicle or 4 μM MHO7 using a Nuclear and Cytoplasmic Protein Extraction Kit (P0028, Beyotime, Shanghai, China) Cells were washed with PBS, scraped off and collected into centrifuge tubes. Cell pellets were resuspended in cytoplasmic extraction buffer A supplemented with PMSF and incubated on ice for 10–15 min. Cytoplasmic extraction buffer B was then added, followed by brief vortexing, incubation on ice for 1 min, and centrifugation at 12,000–16,000× *g* for 5 min at 4 °C. The supernatant was collected as the cytoplasmic fraction. The remaining pellet was resuspended in nuclear extraction buffer supplemented with PMSF, intermittently vortexed on ice for 30 min, and centrifuged at 12,000–16,000× *g* for 10 min at 4 °C. The resulting supernatant was collected as the nuclear fraction. YAP levels were analyzed by Western blotting, with GAPDH and Histone H3 used as cytoplasmic and nuclear markers, respectively.

### 4.11. Xenograft Mouse Model

MDA-MB-231 cells (1.5 × 10^7^ cells/mL) in serum-free RPMI-1640 were subcutaneously inoculated (0.2 mL) into SCID mice. After 2 weeks, tumors with robust growth were aseptically excised, cut into fragments, and cryopreserved at –80 °C. Following a 3-day acclimation period, thawed tumor fragments were rinsed twice in PBS with 1% penicillin-streptomycin, divided into 1–2 mm^3^ blocks, and implanted subcutaneously into the right axilla of 4-week-old female BALB/c-nu mice (16–18 g) via a 0.5 cm incision under isoflurane anesthesia. The protocol was approved by the Laboratory Animal Management and Use Committee of the Medical School, Wuhan University (WAEF-2025-0198; 10 March 2025).

MHO7 nanosuspension was prepared by dissolving 30 mg MHO7 and 7.5 mg SDS in normal saline with zirconia beads, followed by magnetic stirring (1250 rpm, 4 h) to obtain the 6 mg/mL supernatant. When mean tumor volume reached ~120 mm^3^ (2 weeks post-implantation), mice were randomized by tumor size and treated intraperitoneally with saline or MHO7 nanosuspension (40 mg/kg) every other day. Tumor dimensions (length a, width b) and body weight were recorded every other day, and volume was calculated as a × b^2^ × 0.5. After six doses, mice were euthanized and tumors were excised for weighing and documentation.

### 4.12. Immunohistochemistry

Paraffin-embedded tumor sections were deparaffinized and rehydrated, followed by heat-induced epitope retrieval (HIER) in EDTA buffer (pH 9.0) via microwave heating. After cooling, sections were quenched with 3% H_2_O_2_ and blocked with 3% BSA (GC305010, Servicebio, Wuhan, China), then incubated with primary antibodies overnight at 4 °C. The primary antibodies used in this study are detailed in Table S1. Sections were incubated with an HRP-conjugated Goat Anti-Rabbit Secondary Antibody (1:200, GB23303, Servicebio, Wuhan, China) for 50–60 min at room temperature, developed with DAB (3,3′-Diaminobenzidine) (G1212, Servicebio, Wuhan, China), counterstained with hematoxylin, and imaged by bright-field microscopy.

### 4.13. RT-qPCR

Fresh tissues (5–20 mg) were homogenized in 1 mL RNA extraction reagent (G3013, Servicebio, Wuhan, China) using three 3 mm beads in pre-chilled tubes. After chloroform-substitute extraction and phase separation, total RNA was precipitated with pre-cooled isopropanol (–20 °C, 15 min), washed twice with 75% ethanol, air-dried, and resuspended in 15 μL RNase inhibitor–containing resuspension solution. RNA concentration was determined using a NanoDrop 2000 (Thermo Fisher Scientific, Wilmington, DE, USA) and adjusted to 200 ng/μL. cDNA was synthesized by reverse transcription. qPCR was performed using SYBR Green Master Mix (G3326, Servicebio, Wuhan, China). The PCR primer sequences are listed in Table S2. Each cDNA sample was run in triplicate on a real-time PCR system.

### 4.14. Statistical Analysis

Results are expressed as mean ± SD. Normality of data distribution was evaluated prior to statistical testing. For comparisons between two groups, a two-tailed Student’s *t*-test was applied when data followed a normal distribution. Comparisons among three or more groups were analyzed using one-way analysis of variance (ANOVA). When data did not meet normality assumptions, the Kruskal–Wallis test with Dunn’s post hoc correction was used. Differences were considered statistically significant at *p* < 0.05.

## 5. Conclusions

In summary, this study demonstrates that MHO7, a marine-derived fungal metabolite, exerts antitumor activity in ER-positive, tamoxifen-resistant, and triple-negative breast cancer models. MHO7 promotes oxidative stress-associated apoptosis and attenuates YAP/TAZ-TEAD signaling, while NAC intervention attenuated MHO7-induced apoptosis and partially counteracted the MHO7-associated reduction in YAP protein abundance, suggesting that oxidative stress contributes to these responses. These effects are associated with reduced cell viability, wound closure, clonogenic growth, and tumor progression. Collectively, these findings highlight the potential of MHO7 as a lead compound for further investigation in difficult-to-treat breast cancers.

## Data Availability

The data presented in this study are available on request from the corresponding author.

## Supplementary Materials

The following supporting information can be downloaded at https://www.mdpi.com/article/10.3390/md24080273/s1: Figure S1: Fluorescence Intensity Distribution of DCF Signals in LCC2 Cells; Figure S2: Fluorescence Intensity Distribution of DCF Signals in ZR-75-30 Cells; Table S1: Antibodies Used in This Study; Table S2: Primer Sequences Used in This Study; Table S3: Docking Results of MHO7 with the hYAP-hTEAD Complex; Table S4: Docking Results of MHO7 with Palmitoylated TEAD3.

## Abbreviations

The following abbreviations are used in this manuscript:

Annexin V-FITC	fluorescein isothiocyanate-conjugated Annexin V
CCK-8	Cell Counting Kit-8
DCFH-DA	2′,7′-dichlorodihydrofluorescein diacetate
HRP	horseradish peroxidase
IC_50_	half-maximal inhibitory concentration
PBS	phosphate-buffered saline
PI	propidium iodide
TAZ	transcriptional co-activator with PDZ-binding motif
TEAD	TEA domain transcription factor
YAP	yes-associated protein
