# Supplementary material for: Marine-Derived Fungal Metabolite MHO7 Promotes Breast Cancer Apoptosis as a Hippo Pathway Regulator by Modulating the YAP-TEAD Axis
Source: Mar Drugs. 2026 Aug 6;24(8):273. doi: 10.3390/md24080273 (PMC13514928; doi:10.3390/md24080273)
Supplement: Supplementary file 1 [file marinedrugs-24-00273-s001.zip › Supplementary material.pdf]

Supplementary Materials

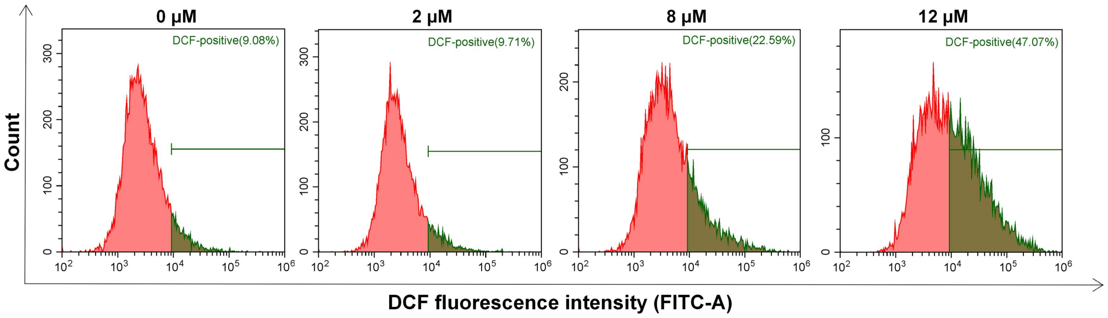

Figure S1. Fluorescence Intensity Distribution of DCF Signals in LCC2 Cells. LCC2 cells were treated with MHO7 (0, 2, 8, and 12 μM) for 48 h and stained with DCFH-DA. The histograms display the distribution of fluorescence intensity. The gated region, labeled as DCF-positive (DCF+), represents the population of cells with elevated intracellular reactive oxygen species (ROS), which oxidize the non-fluorescent DCFH-DA probe into highly fluorescent DCF.

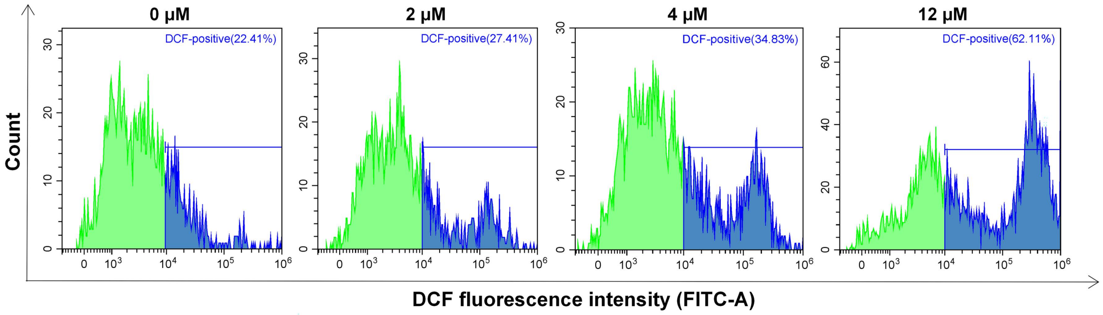

Figure S2. Fluorescence Intensity Distribution of DCF Signals in ZR-75-30 Cells. ZR-75-30 cells were treated with MHO7 (0, 2, 4, and 12 μM) for 48 h and analyzed by flow cytometry. The histograms show a concentration-dependent shift in fluorescence intensity, indicating increased ROS accumulation.

Table S1. Antibodies used in this study

Target	Application	Company	Catalog No.	Host / Type	Dilution	City
YAP	WB	ABclonal	A1002	Rabbit polyclonal	1:2000	Wuhan, China
YAP	IHC-P	ABclonal	A1002	Rabbit polyclonal	1:2000	Wuhan, China
TAZ	WB	ABclonal	A15806	Rabbit polyclonal	1:2000	Wuhan, China
TEAD4	WB	ABclonal	A4151	Rabbit polyclonal	1:2000	Wuhan, China
TEAD4	IHC-P	ABclonal	A23774	Rabbit polyclonal	1:2000	Wuhan, China
Bax	WB	Nature Biosciences	A38131	Rabbit monoclonal antibody	1:2000	Beijing, China
Bcl-2	WB	ABclonal	A19693	Rabbit	1:2000	Wuhan,

				monoclonal antibody		China
Caspase-3	WB	ABclonal	A0214	Rabbit polyclonal	1:2000	Wuhan, China
GAPDH	WB	ABclonal	AC001	Rabbit polyclonal	1:2000	Wuhan, China
CD31	IHC-P	Servicebio	GB113151	Rabbit polyclonal	1:700	Wuhan, China

Table S2. Primer sequences used in this study

**Target gene**	**Primer sequence (5'-3')**
TEAD4	F: TTGGAGTTCTCTGCCTTCCTG
TEAD4	R: GAAGAGATCCTTGAGTCCACCCT
YAP1	F: TGAGAACAATGACGACCAATAGC
YAP1	R: TTCAAGGTAGTCTGGGAAACGG
TAZ	F: CCTCATCACCGTGTCCAATCA
TAZ	R: AGGGGTCCAACGCATCAACT
CD31	F: TTCAACAGAGCCAACCACGC
CD31	R: CTCCGATGATAACCACTGCAATAA
CYR61	F: GAGTGGGTCTGTGACGAGGAT
CYR61	R: AAACAGGGAGCCGCTTCAGT
CTGF	F: AAATGCTGCGAGGAGTGGGT
CTGF	R: TGTCTTCCAGTCGGTAAGCCG
GAPDH	F: GGAAGCTTGTCATCAATGGAAATC
GAPDH	R: TGATGACCCTTTTGGCTCCC

Table S3. Docking results of MHO7 with hYAP-hTEAD complex

**mode**	**Affinity (kcal/mol)**	**Dist from best mode (rmsd l.b.)**	**Dist from best mode (rmsd u.b.)**
1	-4.7	0.000	0.000
2	-4.4	1.356	2.014
3	-4.3	8.082	11.406
4	-4.2	2.914	4.700
5	-4.1	7.590	9.821
6	-3.9	7.668	10.236
7	-3.9	10.123	12.144
8	-3.9	7.693	10.091
9	-3.8	1.842	3.595

Table S4. Docking results of MHO7 with palmitoylated TEAD3

**mode**	**Affinity (kcal/mol)**	**Dist from best mode (rmsd l.b.)**	**Dist from best mode (rmsd u.b.)**
1	-5.7	0.000	0.000
2	-5.5	1.486	2.111

3	-5.3	3.363	8.046
4	-4.1	3.687	8.903
5	-3.9	3.200	5.425
6	-3.7	2.084	2.823
7	-3.6	2.525	3.393
8	-3.1	3.944	8.771
9	-3.1	3.380	5.864
